# Indian Hemp in Facial Neuralgia

**Published:** 1849-07

**Authors:** 


*Indian Hemp in Facial Neuralgia.*—Dr. Rhubaum, of Potsdam, has made several trials of the cannabis indica, in cases of facial neuralgia, with the most satisfactory results. In more than thirty cases great benefit was experienced, and many were entirely cured. Very delicate persons were seized with a little giddiness, lassitute in the limbs, &c.; others were affected in an opposite manner, and evinced great excitement, mirth, and vivacity; but these respective symptoms disappeared after an hour or two, and left no unpleasant sensation behind them. The dose was from sixteen to twenty drops of the tincture, containing about one grain of the resinous extract.—*Medicinische Zeitung.*

